# Effectiveness of telemedicine for pregnant women with gestational diabetes mellitus: an updated meta-analysis of 32 randomized controlled trials with trial sequential analysis

**DOI:** 10.1186/s12884-020-02892-1

**Published:** 2020-04-06

**Authors:** Weihua Xie, Pinyuan Dai, Yu Qin, Ming Wu, Bingquan Yang, Xiaojin Yu

**Affiliations:** 1 Department of Epidemiology and Health Statistics, School of Public Health, Southeast University, Nanjing, China; 2 Jiangsu Provincial Center for Disease Control and Prevention, Nanjing, China; 3 Zhongda Hospital, Southeast University, Nanjing, China

**Keywords:** Gestational diabetes mellitus, Telemedicine, Systematic review, Meta-analysis, Trial sequential analysis

## Abstract

**Background:**

Gestational diabetes mellitus (GDM) is now a global health problem. Poor blood glucose control during pregnancy may lead to maternal and neonatal/foetal complications. Recently, the development of information and communication technology has resulted in new technical support for the clinical care of GDM. Telemedicine is defined as health services and medical activities provided by healthcare professionals through remote communication technologies. This study aimed to update the systematic review of the effectiveness of telemedicine interventions on glycaemic control and pregnancy outcomes in pregnant women with GDM.

**Methods:**

We searched the Web of Science, PubMed, Scopus, Cochrane Central Register of Controlled Trials, Chinese National Knowledge Infrastructure, Wan-fang Database, China Biology Medicine and VIP Database for randomized controlled trials (RCTs) related to the effectiveness of telemedicine interventions for GDM from database inception to July 31st, 2019. Languages were limited to English and Chinese. Literature screening, data extraction and assessment of the risk of bias were completed independently by two reviewers. Meta-analysis and trial sequential analysis were conducted in Stata 14.0 and TSA v0.9.5.10 beta, respectively.

**Results:**

A total of 32 RCTs were identified, with a total of 5108 patients. The meta-analysis showed that telemedicine group had significant improvements in controlling glycated haemoglobin (HbA1c) [mean difference (MD) = − 0.70, *P* < 0.01], fasting blood glucose (FBG) (MD = -0.52, *P* < 0.01) and 2-h postprandial blood glucose (2hBG) (MD = -1.03, *P* = 0.01) compared to the corresponding parameters in the standard care group. In the telemedicine group, lower incidences of caesarean section [relative risk (RR) = 0.82, *P* = 0.02], neonatal hypoglycaemia (*RR* = 0.67, *P* < 0.01), premature rupture of membranes (*RR* = 0.61, *P* < 0.01), macrosomia (*RR* = 0.49, *P* < 0.01), pregnancy-induced hypertension or preeclampsia (RR = 0.48, *P* < 0.01), preterm birth (*RR* = 0.27, *P* < 0.01), neonatal asphyxia (*RR* = 0.17, *P* < 0.01), and polyhydramnios (*RR* = 0.16, *P* < 0.01) were found. The trial sequential analyses conclusively demonstrated that the meta-analytic results of the change in HbA1c, the change in 2hBG, the change in FBG, the incidence rates of caesarean section, pregnancy-induced hypertension or preeclampsia, premature rupture of membranes, premature birth, neonatal asphyxia, and polyhydramnios were stable.

**Conclusions:**

Compared to standard care, telemedicine interventions can decrease the glycaemic levels of patients with GDM more effectively and reduce the risk of maternal and neonatal/foetal complications.

## Background

Gestational diabetes mellitus (GDM) is a common complication during pregnancy. According to the report of the International Diabetes Federation, the global prevalence of GDM in 2017 has reached 14.0%, affecting nearly 21.3 million live births [1]. Johns et al. [2] reported that poor glycaemic control during pregnancy would increase the risk of maternal and neonatal/foetal complications, such as neonatal hypoglycaemia, macrosomia, preeclampsia, preterm birth, and polyhydramnios. Therefore, the blood glucose dynamics of pregnant women with GDM should be promptly reported to healthcare professionals for scientific guidance. Currently, the standard care practice for patients with GDM is that the pregnant women monitor the glycaemic levels and record by hand in paper diaries several times per day at home and then healthcare professionals review the glycaemic data and provide health education during the regular antenatal examination or face-to-face consultation [3–5]. The traditional mode of standard care has some shortcomings, such as lagging information and insufficient communication between doctors and patients. Recently, the rapid development of information and communication technology provides new technical support and management modes for improving the clinical care of patients [6–13]. Telemedicine (TM) refers to health services and medical activities, such as the remote evaluation, diagnosis and treatment of patients by healthcare professionals performed using remote communication technologies, such as mobile phones, Bluetooth, telephones, email, and websites. More specifically, healthcare professionals monitor patients’ health-related indicators and provide timely medical feedback through website-based systems or mobile terminal devices, and remotely provide health knowledge and guidance to improve the physical and psychological status of patients [14, 15]. Ideally, TM facilitates the clinical management of diabetes by uploading glucose data, symptoms and signs in real-time and providing medical consultation and health education, which offers great convenience for patients in remote areas.

Most of the systematic reviews and meta-analyses on the use of telemedicine technologies for diabetes mellitus have demonstrated that TM tools could result in reduced HbA1c in individuals with type I, type II diabetes and GDM [8, 9, 13, 14, 16]. However, the effects of TM on other indices of glucose and pregnancy outcomes of the use of TM in patients with GDM remain uncertain [14, 17]. Rasekaba et al. [17] performed a meta-analysis of 3 RCTs comparing TM to standard care that included 228 pregnant women in 2015; the meta-analysis revealed no beneficial impacts of TM on glycated haemoglobin (HbA1c), 2-h postprandial blood glucose (2hBG) or the incidence of caesarean section. Moreover, the meta-analysis conducted by Ming et al. [14] revealed significant improvement in HbA1c and no differences in other maternal and neonatal outcomes between the TM group and the standard care group. In the last few years, some more studies that demonstrate the effects of TM in patients with GDM have been conducted. Guo et al. [6] reported that TM intervention could reduce the level of HbA1c more effectively but that it had no significant effect on fasting blood glucose (FBG) and 2hBG. Miremberg et al. [4] showed that mean blood glucose was reduced through the use of a TM intervention and no significant reduction in the incidence of pregnancy complications was reported. Nevertheless, some recent studies have also indicated the advantages of TM in the context of blood glucose [3, 18, 19] and the incidence of caesarean section [7], preterm birth [20, 21], premature rupture of membranes [22], macrosomia [23] and neonatal hypoglycaemia [19, 24].

Given the inconsistencies among the results from recent studies, an updated meta-analysis with trial sequential analysis (TSA) was conducted to compare TM interventions with standard care in pregnant women with GDM. The aims of this study were evaluating the effectiveness of TM with the standard care in pregnant women with GDM, comparing the effects of different ways of TM, and selecting a better management mode for GDM. Subgroup analyses of studies according to the types of TM tools and the patterns of TM interventions were also carried out in this study to explore the potential source of the heterogeneity between studies and provide the estimates of effects for different ways of TM interventions.

## Methods

### Search strategy and selection criteria

We followed the Preferred Reporting Items for Systematic Reviews and Meta-Analysis (PRISMA) guidelines to perform the systematic review and meta-analysis [25] (completed PRISMA checklist is provided in Additional file 1). The Web of Science, PubMed, Scopus, Cochrane Central Register of Controlled Trials (CENTRAL), Chinese National Knowledge Infrastructure (CNKI), Wan-fang Database, China Biology Medicine (CBM) and VIP Database were used to search for relevant randomized controlled trials (RCTs) comparing TM to standard care from database inception to July 31st, 2019. We limited the language to English and Chinese. The search terms consisted of telemedicine-related terms (mobile OR digital OR mhealth OR m-health OR ehealth OR e-health OR app OR apps OR application* OR telemedicine OR tele-medicine OR smartphone OR smart phone OR cell phone OR telehealth OR tele-health OR tele-care OR telecare OR electronic* OR web-based OR technolog* OR short messag* OR SMS OR text message* OR texting OR remote OR internet OR WeChat OR QQ) and GDM-related terms (pregnan* diabet* OR gestation* diabet* OR pregnan* hypergly* OR gestation* hypergly* OR GDM). We also searched the reference lists of the identified studies. The Web of Science search strategy is listed in Additional file 2.

### Inclusion criteria

Studies meeting the following criteria were included: (a) complete RCTs that enrolled pregnant women with GDM, (b) trials in which the patients in the trial group received the TM interventions and those in the control group received standard care, and (c) studies published in English or Chinese.

### Exclusion criteria

Studies were excluded if (a) they were duplicate publications, literature reviews or meta-analyses, (b) they enrolled women with other types of diabetes, such as type I diabetes and type II diabetes, (c) they were non-randomized controlled trials, or (d) they had insufficient data for extraction.

### Outcomes

The primary outcomes were the indicators of maternal glycaemic control during pregnancy, such as (1) glycated haemoglobin (HbA1c, %), a form of haemoglobin that is bound to glucose, which reflects the average level of blood glucose over the past 2 to 3 months; (2) fasting blood glucose (FBG, mmol/L), the blood glucose level after fasting or not eating anything for at least 8 h; and (3) 2-h postprandial blood glucose (2hBG, mmol/L), the blood glucose level measured exactly 2 h after eating a meal. In this study, we calculated the difference in HbA1c, FBG, and 2hBG before and after the intervention as the change in HbA1c, FBG, and 2hBG.

The secondary outcomes were maternal and neonatal/foetal complications, including the incidence of caesarean section, pregnancy-induced hypertension (PIH) or preeclampsia, premature rupture of membranes, macrosomia, admission to the neonatal intensive care unit (NICU), neonatal jaundice or hyperbilirubinemia, neonatal acute respiratory distress syndrome (NARDS), neonatal hypoglycaemia, preterm birth, neonatal asphyxia, and polyhydramnios.

### Study selection, data extraction, and quality assessment

Two reviewers (WX and YQ) independently screened the literature, extracted the data and assessed the risk of bias of the included research. If there was any inconsistency, a decision was made by discussion. When screening the literature, we first excluded the obviously ineligible literature by reading the title and abstract, and then further read the full text to identify the studies to be included. The data extracted mainly comprised (1) the basic characteristics of the literature: the first author, the year of publication, the country or region, the sample size, etc., (2) the outcome indicators and related data, and (3) and the risk of bias of the study, which was evaluated by using the Cochrane Collaboration Risk of Bias Tool [26, 27], and included bias related to random sequence generation, allocation concealment, blinding of participants and personnel, blinding of outcome assessment, incomplete outcome data, selective reporting, and other bias.

### Data synthesis and analysis

Meta-analysis was performed by Stata software (version 14, StataCorp, College Station, USA). The mean difference (MD) with the 95% confidence interval (CI) is presented for continuous data, where MD reflects the absolute difference between the average value in two groups in a clinical trial and estimates the average amount by which the trial intervention changed the outcome on average compared to the outcome of the control. The relative risk (RR) with the 95% CI was calculated for binary categorical data, where RR is the ratio of the probability of a certain outcome occurring in two different groups and it describes the multiplication of the risk that occurs with the use of the trial intervention. Here, the smaller value of RR means a larger effect size for the TM interventions in reducing the risk of complication outcomes. The cut-off values of RR for small, medium and large effects are 0.82, 0.54 and 0.33 respectively [28].

The heterogeneity among the results was analysed by the Q-test (significance level α = 0.10) and quantitatively judged by the I^2^ statistic, where I^2^ > 40% was considered to be evidence of substantial heterogeneity. If the heterogeneity among the results was statistically significant, the source of heterogeneity was further analysed and the random effect model was used for meta-analysis. If there was no significant heterogeneity among the results, the fixed-effect model was used. The sources of heterogeneity were explored by meta-regression (significance level α = 0.10). Subgroup analysis was conducted to explore the differences between different types of TM tools and patterns of TM interventions. Sensitivity analysis was conducted to assess the robustness of the pooled results after a study with high risk was removed. The significance level of the meta-analysis was set to 0.05. Moreover, funnel plots were used to evaluate publication bias. If the funnel plot is asymmetric, it suggests that there may be publication bias. Moreover, we used the standard deviation of the change to quantify the amount of variation in the changes in glycaemic levels (HbA1c, FBG, and 2hBG). The standard deviations of the change were calculated by the following formula:

$$ {\mathrm{S}}_{\mathrm{d}}=\sqrt{{\mathrm{S}}_1^2+{\mathrm{S}}_2^2-2\times \mathrm{r}\times {\mathrm{S}}_1\times {\mathrm{S}}_2} $$

where S_1_ and S_2_ denote the standard deviation of pre- and post-intervention, respectively. The correlation coefficient (r) between measurements of pre- and post-intervention was set to 0.5 in this study.

The trial sequential analysis (TSA) was carried out by using TSA software (version v0.9.5.10 beta, Copenhagen Trial Unit, Copenhagen, Denmark). In this study, the relative risk reduction (RRR), level of type I error and level of type II error were set to 20%, 0.05 and 0.20, respectively, to calculate the required information size (RIS). The monitoring boundary and futility boundary are presented to assess the evidence provided by each study sequentially. When the total sample size of the studies reaches the RIS or the cumulative Z-value surpasses the monitoring boundary or futility boundary, it concludes that there is sufficient evidence regarding the effects of TM interventions from the meta-analysis.

## Results

### Study selection process and basic characteristics of the included studies

**Fig. 1:**
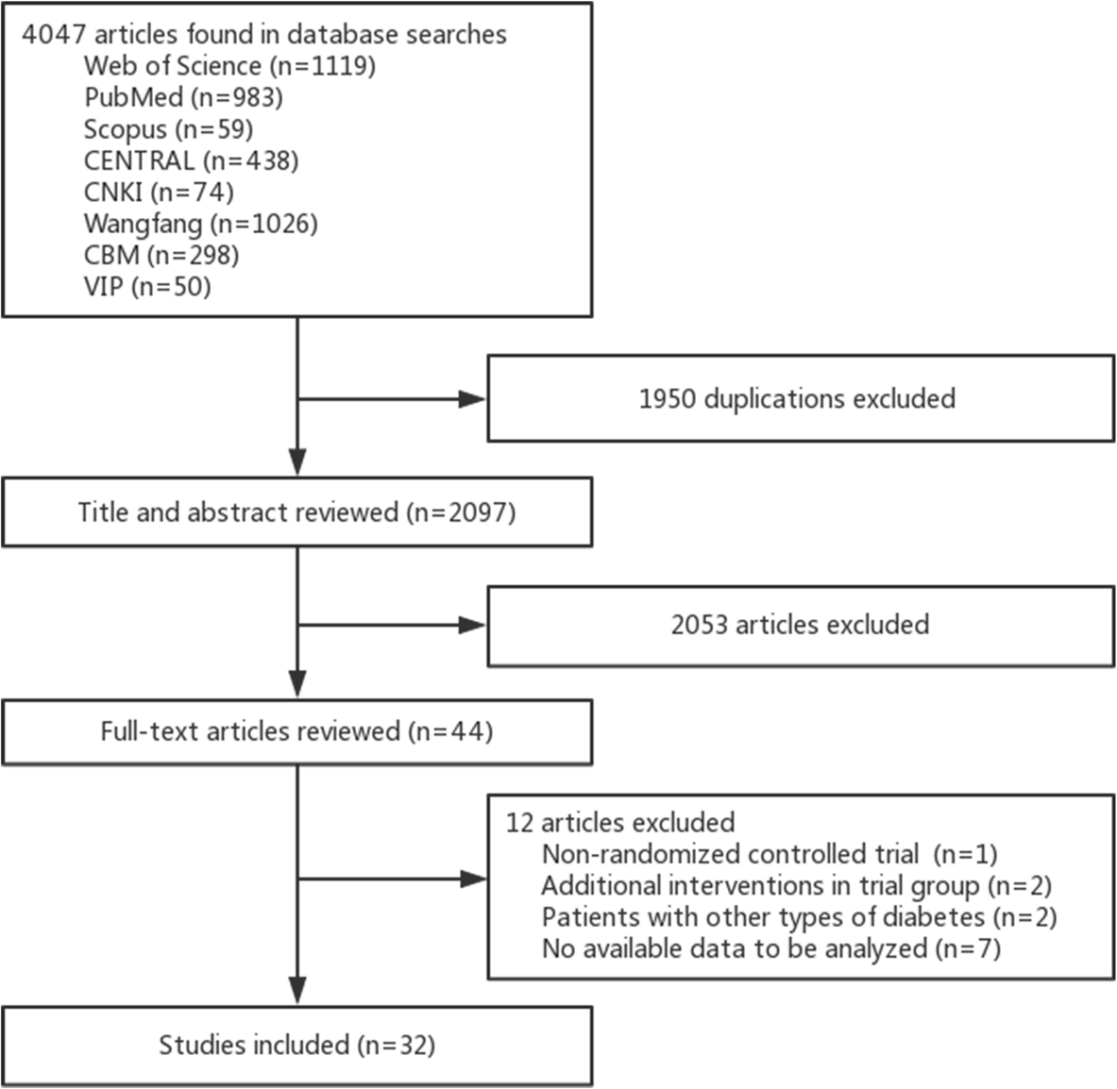
Flow chart of the study selection process

We initially identified 4047 publications from eight electronic databases. After screening the titles and abstracts and reviewing full texts, 32 RCTs were included: 13 English and 19 Chinese papers. The selection process is shown in Fig. 1.

A total of 5108 patients with GDM were included in this study, including the trial group (*n* = 2581) and the control group (*n* = 2527). Most studies were conducted in China (21 studies, 65.6%). The sample size ranged from 44 to 820. TM interventions mainly consisted of web-based systems (7 trials), health devices (3 trials), health apps (7 trials), and WeChat (15 trials) (detailed descriptions are shown in Additional file 3**)**. The basic characteristics and outcomes of each included study are listed in Table 1.

**Table 1:** Basic characteristics and outcomes of included studies

Study	Country/region	Sample size (Trial/Control)	TM intervention^a^	Mean age (year)	College education (%)	Gestational age at enrolment (week)	Outcome^b^
Carral 2015 [29]	Spain	77 (30/47)	Web-based system	33.8	22.1	21.1	①
Dalfra 2009 [30]	Italy	203 (88/115)	Health device	34.0	–	28.0	①④⑦
Given 2015 [31]	North Ireland and Ireland	50 (24/26)	Health device	31.7	–	28.0	④⑦⑧⑨⑩⑫
Guo 2019 [6]	China	124 (64/60)	Health app	30.9	55.6	24.9	①②③④⑦⑪
Homko 2007 [32]	USA	57 (32/25)	Web-based system	29.5	43.9	27.6	②④⑤⑥⑧⑨⑩⑪⑫
Homko 2012 [33]	USA	80 (40/40)	Web-based system	30.2	60.0	28.5	②④⑤⑥⑧⑨⑩⑪⑫
Mackillop 2018 [7]	UK	203 (101/102)	Health app	33.5	48.5	31.0	②④⑤⑦⑧⑨⑪⑫
Miremberg 2018 [4]	Israel	120 (60/60)	Health app	31.9	32.5	–	④⑤⑧⑩⑪⑭
Perez-Ferre 2010 [34]	Spain	97 (49/48)	Health app	33.8	15.5	–	④⑤⑪⑫
Rasekaba 2018 [35]	Australia	95 (61/34)	Web-based system	32.0	–	28.0	②③④⑦⑧
Yang 2018 [36]	China	107 (57/50)	WeChat	31.9	–	–	②③④⑤⑥⑦⑧⑨⑪⑫
Carolan-Olah 2019 [37]	Australia	110 (52/58)	Web-based system	31.7	47.3	28–30	⑦
Kim 2019 [3]	Korea	44 (22/22)	Web-based system	35.8	97.7	27.4	①②
Gao 2017 [18]	China	58 (28/30)	Health app	27.8	31.0	–	①②③
Hua 2018 [19]	China	120 (60/60)	WeChat	27.6	25.8	24.0	①②③④⑦⑪⑫
Zhang 2018 [38]	China	80 (40/40)	Health app	29.5	–	26.9	②③
Zhao 2018 [39]	China	60 (30/30)	Health app	–	–	–	②③
Zeng 2017 [40]	China	86 (43/43)	WeChat	–	74.4	–	④⑪
Fang 2017 [41]	China	60 (30/30)	WeChat	30.8	–	23.0	①②③④⑥⑦⑨⑪⑫⑬
Ge 2017 [23]	China	586 (308/278)	WeChat	30.5	45.1	26.0	④⑦
Huang 2016 [42]	China	80 (40/40)	WeChat	39.0	28.8	36.7	①②③④
Jiang 2017 [43]	China	150 (78/72)	WeChat	28.0	–	29.5	①②③④⑤⑦⑩⑪⑭
Jiang 2019 [44]	China	200 (100/100)	WeChat	28.7	–	–	④⑤⑦⑫⑬⑭
Jiang 2016 [20]	China	120 (60/60)	WeChat	25.3	–	22.8	⑤⑦⑫⑭
Liu 2018 [45]	China	98 (49/49)	Health device	27.8	–	30.3	①②③
Lu 201 [21]	China	280 (140/140)	WeChat	26.1	47.9	–	①②③⑤⑦⑫⑬⑭
Luo 2017 [46]	China	107 (57/50)	WeChat	31.9	–	–	②④⑥
Shao 2018 [47]	China	410 (205/205)	WeChat	30.5	52.9	–	②③⑦
Su 2018 [22]	China	820 (420/400)	Web-based system	28.2	27.0	–	②③⑤⑥⑦⑫⑬⑭
Weng 2018 [48]	China	120 (60/60)	WeChat	39.5	53.3	36.6	①②③④
Xiao 2016 [24]	China	206 (103/103)	WeChat	29.7	–	26.4	⑦⑪
Yang 2015 [49]	China	100 (50/50)	WeChat	30.0	80.0	–	⑤⑥⑦⑫⑬⑭

^a^TM intervention: Detailed descriptions are listed in Additional file 3. ^b^Outcome: ①change of HbA1c; ②change in FBG; ③change in 2hBG; ④caesarean section; ⑤pregnancy induced hypertension or preeclampsia; ⑥premature rupture of membranes; ⑦macrosomia; ⑧admission to the NICU; ⑨neonatal jaundice or hyperbilirubinemia; ⑩NARDS; ⑪neonatal hypoglycaemia; ⑫preterm birth; ⑬neonatal asphyxia; ⑭polyhydramnios

### Assessment of the risk of bias

**Fig. 2:**
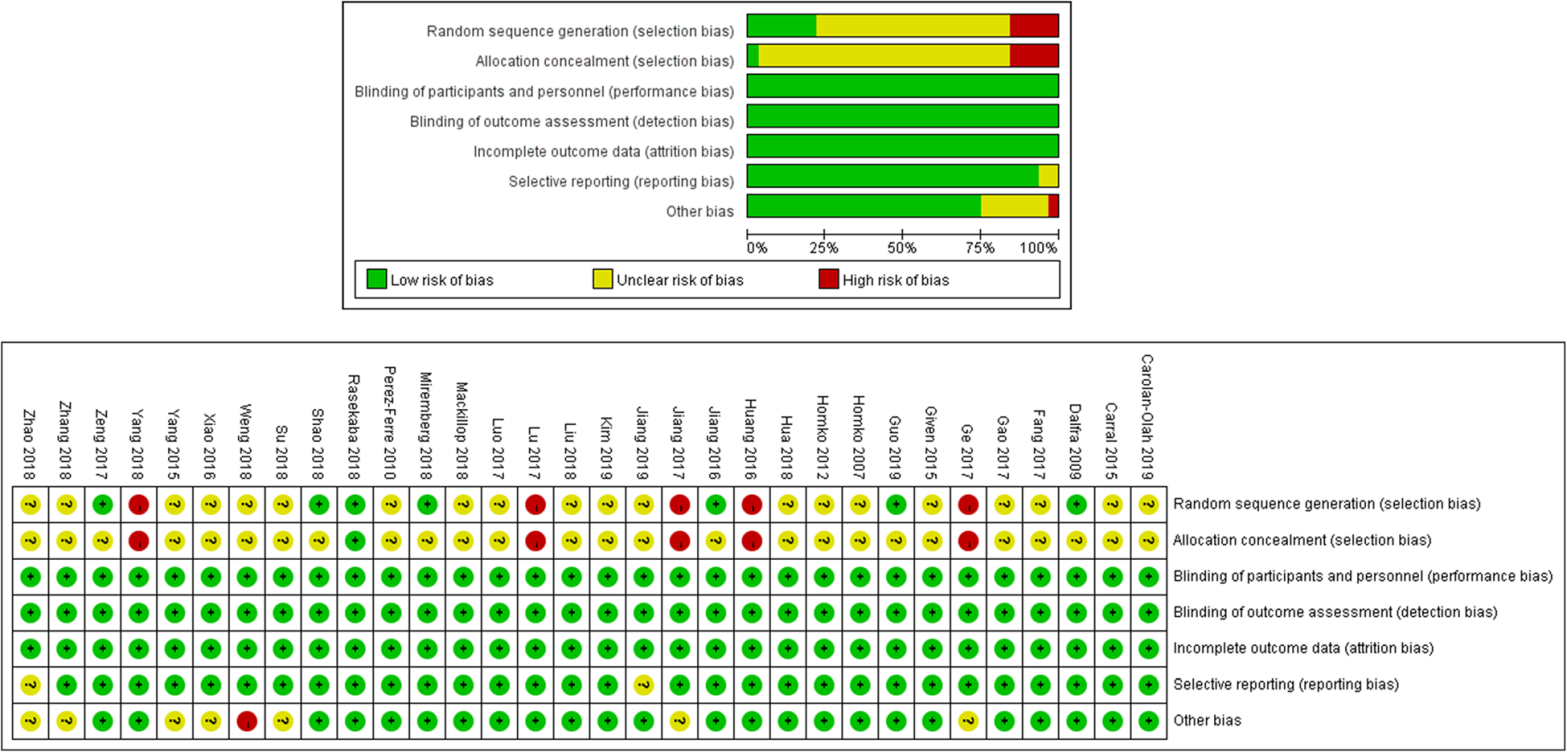
Assessment of risk of bias

Methods of random sequence generation and allocation concealment were not clearly described in most of the included studies. Only 12 out of 32 studies described the methods of random sequence generation and six trials described allocation concealment, which may cause a high risk of bias in this study. The overall summary and individual risk of bias are given at length in Fig. 2.

### Glycaemic control

**Fig. 3:**
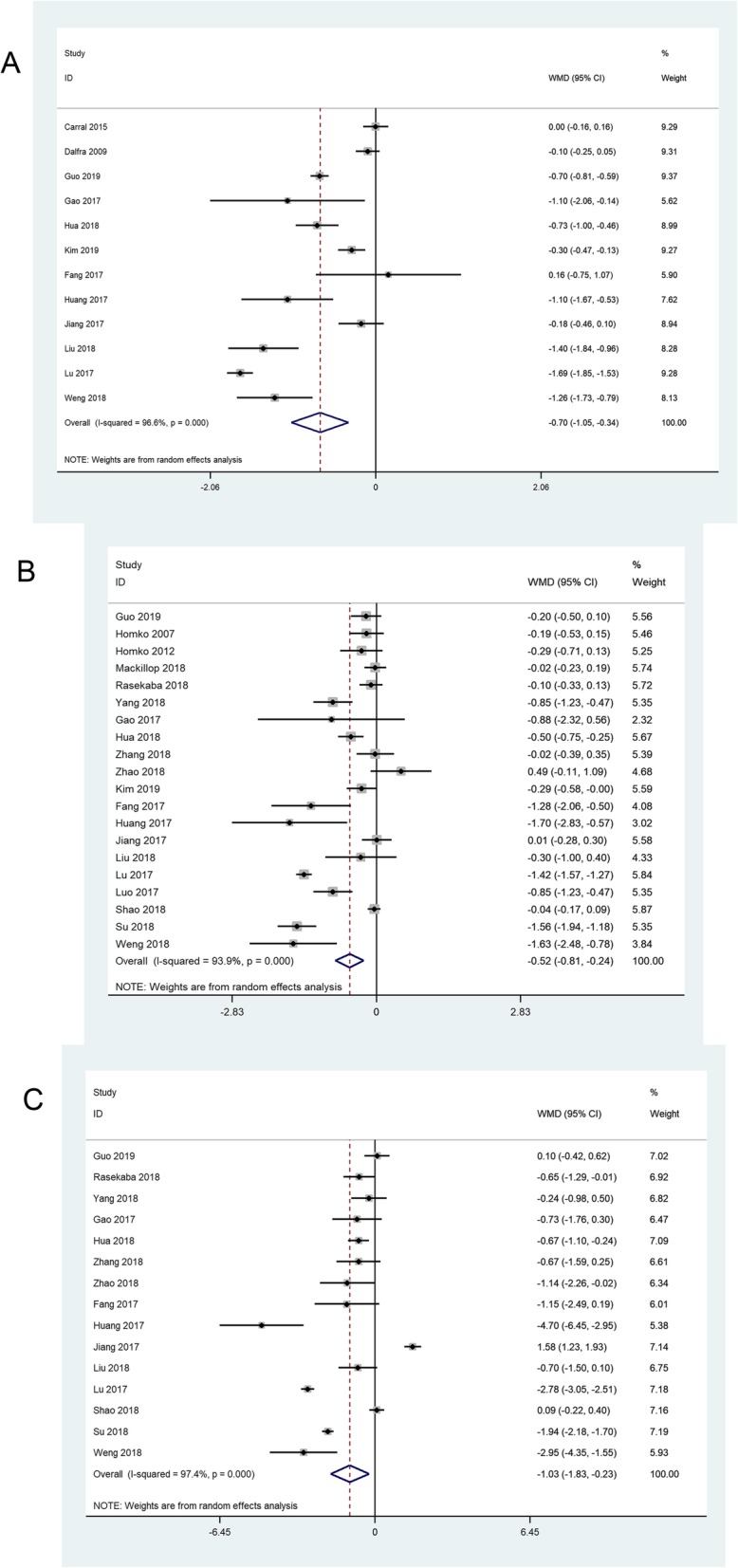
The meta-analyses of the change in HbA1c (**a**), FBG (**b**) and 2hBG (**c**)

The meta-analysis of 12 trials [3, 6, 18, 19, 21, 29, 30, 41–43, 45, 48] showed that the change in HbA1c in the TM group was higher than that in the control group [MD = -0.70, 95% CI = (− 1.05, − 0.34), *P* < 0.01] with high heterogeneity (I^2^ = 97%, *P* < 0.01) (Fig. 3a). The sensitivity analysis demonstrated that the pooled effect and I^2^ statistic changed minimally after item-by-item exclusion. Meta-regression analysis showed that the gestational age at enrolment (β = − 0.07, *P* = 0.05) and the location of the study (β = 0.79, *P* = 0.03) might be the reasons for the high heterogeneity of the change in HbA1c.

The pooled effect of 20 trials [[3, 6, 7, 18, 19, 21, 22, 32, 33, 35, 36, 38, 39, 41–43, 45–48] revealed a significant advantage in the TM group compared with the standard care group in regard to the change in FBG [MD = -0.52, 95% CI = (− 0.81, − 0.24), *P* < 0.01] (Fig. 3b). Nevertheless, there was an obvious heterogeneity (I^2^ = 94%, *P* < 0.01) among studies. Sensitivity analysis showed the robustness of pooled effect and meta-regression did not reveal the source of heterogeneity.

The result of the meta-analysis of 15 studies [6, 18, 19, 21, 22, 35, 36, 38, 39, 41–43, 45, 47, 48] indicated greater change in 2hBG in the TM group than that in the control group [MD = -1.03, 95% CI = (− 1.83, − 0.23), *P* = 0.01] (Fig. 3c). Significant inter-study heterogeneity (I^2^ = 97%, *P* < 0.01) was observed, and the average age of pregnant women (β = − 0.19, *P* = 0.08) and the gestational age at enrolment (β = − 0.22, *P* = 0.07) were considered sources of heterogeneity according to the meta-regression.

### Maternal and neonatal/foetal complications

Data from 19 trials [4, 6, 7, 19, 23, 30–32, 34–36, 40–44, 46, 48] involving 2374 pregnant women reported that TM interventions played a significant role in decreasing the incidence of the caesarean section when compared to the control condition [*RR* = 0.82, 95% CI = (0.69, 0.97), *P* = 0.02] with mildly significant heterogeneity among studies (I^2^ = 57%, *P* < 0.01) (Table 2). Sensitivity analysis revealed that the pooled effect on caesarean section was mainly influenced by three trials [32, 36, 46], but the specific sources of heterogeneity were not identified in the meta-regression. After excluding these three trials, the pooled RR and I^2^ statistic dropped to 0.72 (*P* < 0.01) and 15%, respectively.

**Table 2:** Meta-analyses of maternal/foetal outcomes of TM interventions compared to standard care in patients with GDM

Outcome	No. of trials	Pooled effect			Heterogeneity	
		Effect size	95%CI	P	I^2^	P
Caesarean section	19 [4, 6, 7, 19, 23, 30–32, 34–36, 40–44, 46, 48]	RR = 0.82	(0.69, 0.97)	0.02	57	0.01
PIH or preeclampsia	12 [4, 7, 20–22, 32–34, 36, 43, 44, 49]	RR = 0.48	(0.40, 0.58)	< 0.01	11	0.34
Premature rupture of membranes	7 [22, 32, 33, 36, 41, 46, 49]	RR = 0.61	(0.50, 0.76)	< 0.01	15	0.31
Macrosomia	18 [6, 7, 19–24, 30, 31, 35–37, 41, 43, 44, 47, 49]	RR = 0.49	(0.30, 0.80)	< 0.01	77	< 0.01
Neonatal hypoglycaemia	12 [4, 6, 7, 19, 24, 32–34, 36, 40, 41, 43]	RR = 0.67	(0.51, 0.87)	< 0.01	39	0.08
Preterm birth	13 [7, 19–22, 31–34, 36, 41, 44, 49]	RR = 0.27	(0.20, 0.35)	< 0.01	38	0.08
Neonatal asphyxia	5 [21, 22, 41, 44, 49]	RR = 0.17	(0.08, 0.33)	< 0.01	0	0.85
Polyhydramnios	7 [4, 20–22, 43, 44, 49]	RR = 0.16	(0.10, 0.28)	< 0.01	0	0.99
Admission to the NICU	7 [4, 7, 31–33, 35, 36]	RR = 0.89	(0.60, 1.32)	0.57	20	0.28
Neonatal jaundice or hyperbilirubinemia	6 [7, 31–33, 36, 41]	RR = 1.00	(0.64, 1.55)	0.99	0	0.68
NARDS	6 [7, 31–33, 36, 41]	RR = 0.66	(0.33, 1.33)	0.25	0	0.41

*GDM* Gestational diabetes mellitus, *PIH* pregnancy-induced hypertension, *NICU* neonatal intensive care unit, *NARDS* neonatal acute respiratory distress syndrome, *RR* relative risk, *CI* confidence interval, *TM* telemedicine

The pooled RR of 12 studies [4, 7, 20–22, 32–34, 36, 43, 44, 49] using a fixed effect model showed that the incidence of PIH or preeclampsia in the TM group was significantly lower than that in the control group [*RR* = 0.48, 95% CI = (0.40, 0.58), *P* < 0.01; I^2^ = 11%, *P* = 0.34].

The results of meta-analysis of 7 trials [22, 32, 33, 36, 41, 46, 49] using a fixed-effect model showed that the incidence of premature rupture of membranes in the TM group was significantly lower than that in the control group [*RR* = 0.61, 95% CI = (0.50, 0.76), *P* < 0.01], with insignificant heterogeneity among studies (I^2^ = 15%, *P* = 0.31).

The pooled result of eighteen studies [6, 7, 19–24, 30, 31, 35–37, 41, 43, 44, 47, 49] confirmed that TM interventions could significantly reduce the risk of macrosomia compared to the standard care [*RR* = 0.49, 95% CI = (0.30, 0.80), *P* < 0.01], with substantial heterogeneity (I^2^ = 77, *P* < 0.01). Two trials [7, 22] were identified as sources of heterogeneity by sensitivity analysis. In comparison to the rest of the included studies, the study conducted by Mackillop et al. [7] included patients with an older age (33.5 years old) and higher gestational age at enrolment (31 weeks) on average, and Su et al’s trial [22] had the largest number of patients (*n* = 820). The re-pooled results showed no significant heterogeneity after removing these two trials at the same time (I^2^ = 27%, *P* = 0.15). Furthermore, meta-regression illustrated that the heterogeneity among studies might be caused by sample size (β = − 0.01, *P* = 0.06), the location of study (β = 1.42, *P* < 0.01), the average age of pregnant women (β = 0.26, *P* = 0.02), gestational age at enrolment (β = 0.15, *P* = 0.04) and the proportion of primipara (β = − 5.39, *P* = 0.08).

The results of a meta-analysis of 12 trials [4, 6, 7, 19, 24, 32–34, 36, 40, 41, 43] using a random effect model showed that the incidence of neonatal hypoglycaemia in the TM group was significantly lower than that in the control group [*RR* = 0.67, 95% CI = (0.51, 0.87), *P* < 0.01; I^2^ = 39%, *P* = 0.08]. In the meta-analysis of 13 trials [7, 19–22, 31–34, 36, 41, 44, 49], there was a significantly lower risk of preterm birth in the TM group than in the standard care group [*RR* = 0.27, 95% CI = (0.20, 0.35), *P* < 0.01; I^2^ = 38%, *P* = 0.08].

The meta-analysis of five studies [21, 22, 41, 44, 49] found sufficient evidence of a beneficial effect of TM interventions on the incidence of neonatal asphyxia [*RR* = 0.17, 95% CI = (0.08, 0.33), *P* < 0.01], and no significant heterogeneity was present among these studies (I^2^ = 0, *P* = 0.85). The pooled effect of 7 trials [4, 20–22, 43, 44, 49] indicated that there was significant reduction in the risk of polyhydramnios in the TM group [*RR* = 0.16, 95% CI = (0.10, 0.28), *P* < 0.01], without the existence of heterogeneity (I^2^ = 0, *P* = 0.99).

The overall result of the meta-analysis demonstrated no significant reduction in the risk of the incidence of admission to the NICU [4, 7, 31–33, 35, 36], neonatal jaundice or hyperbilirubinemia [7, 31–33, 36, 41], or NARDS [7, 31–33, 36, 41] between the two groups [*RR* = 0.89, 95% CI = (0.60, 1.32), *P* = 0.57; RR = 1.00, 95% CI = (0.64, 1.55), P = 0.99; *RR* = 0.66, 95% CI = (0.33, 1.33), *P* = 0.25, respectively], without significant heterogeneity (I^2^ = 20%, *P* = 0.28; I^2^ = 0, *P* = 0.68; I^2^ = 0, *P* = 0.41, respectively), (Table 2).

### Subgroup analysis

For the subgroup analysis, we identified three subgroups for each outcome indicator corresponding to three types of TM tools, including health app or device, web-based system, and WeChat (Table 3). Only the subgroups with more than 2 trials were analysed. Patients receiving WeChat interventions were considered to benefit more than those receiving interventions using health app or device and web-based system in regard to the change in HbA1c, the change in FBG, and the incidence of PIH or preeclampsia, macrosomia, and neonatal hypoglycaemia. The health app or device subgroup exhibited a greater reduction in the incidence of caesarean section than the web-based system and WeChat. Compared with standard care, health app or device could significantly reduce 2hBG, but no significant difference was found in the WeChat subgroup. Regarding the incidence of premature rupture of membranes and the incidence of preterm birth, the web-based system reduced the risk more effectively than health app or device and WeChat. Furthermore, low heterogeneity existed in the subgroup of health app or device on the change in FBG, the change in 2hBG, the incidence of caesarean section and macrosomia, and little heterogeneity (I^2^ = 4%) was found in the subgroup analysis of the effect of WeChat on the incidence of macrosomia.

**Table 3:** Subgroup analyses of studies using different types of TM tools

Outcome	TM tools	No. of trials	Pooled effect			Heterogeneity	
			Effect size	95% CI	P	I^2^	P
Change in HbA1c	Health app or device	4	MD = -0.75	(− 1.25, − 0.25)	< 0.01	95	0.01
	WeChat	6	MD = -0.84	(−1.46, − 0.22)	< 0.01	95	< 0.01
Change in FBG	Health app or device	6	MD = -0.05	(−0.23, 0.12)	0.54	16	0.31
	Web-based system	5	MD = -0.48	(− 0.95, − 0.00)	0.05	91	< 0.01
	WeChat	9	MD = -0.85	(−1.35, −0.35)	< 0.01	96	< 0.01
Change in 2hBG	Health app or device	5	MD = -0.50	(−0.97, − 0.03)	0.04	38	0.17
	WeChat	8	MD = -1.26	(−2.63, 0.11)	0.07	98	< 0.01
Caesarean section	Health app or device	6	RR = 0.76	(0.60, 0.97)	0.03	33	0.19
	Web-based system	3	RR = 1.19	(0.69, 2.06)	0.54	67	0.05
	WeChat	10	RR = 0.82	(0.60, 0.96)	0.02	56	0.02
PIH or preeclampsia	Health app or device	3	RR = 0.76	(0.28, 2.09)	0.60	35	0.22
	Web-based system	3	RR = 0.50	(0.40, 0.62)	< 0.01	70	0.04
	WeChat	6	RR = 0.39	(0.26, 0.61)	< 0.01	0	0.93
Premature rupture of membranes	Web-based system	3	RR = 0.56	(0.44, 0.71)	< 0.01	21	0.28
	WeChat	3	RR = 0.80	(0.53, 1.19)	0.27	0	0.51
Macrosomia	Health app or device	4	RR = 1.16	(0.65, 2.06)	0.62	27	0.25
	Web-based system	3	RR = 0.61	(0.07, 5.58)	0.66	81	< 0.01
	WeChat	11	RR = 0.44	(0.32, 0.59)	< 0.01	4	0.40
Admission to the NICU	Health app or device	3	RR = 0.74	(0.44, 1.24)	0.25	14	0.32
	Web-based system	3	RR = 1.33	(0.69, 2.59)	0.40	46	0.16
Neonatal hypoglycaemia	Health app or device	4	RR = 1.25	(0.81, 1.92)	0.31	0	0.78
	WeChat	6	RR = 0.40	(0.28, 0.59)	< 0.01	0	0.86
Preterm birth	Health app or device	3	RR = 0.40	(0.17, 0.96)	0.04	0	0.75
	Web-based system	3	RR = 0.22	(0.15, 0.32)	< 0.01	84	< 0.01
	WeChat	7	RR = 0.32	(0.21, 0.48)	< 0.01	0	0.66
Neonatal asphyxia	WeChat	4	RR = 0.17	(0.08, 0.33)	< 0.01	0	0.88
Polyhydramnios	WeChat	5	RR = 0.17	(0.08, 0.35)	< 0.01	0	0.99

*GDM* Gestational diabetes mellitus, *HbA1c* glycated haemoglobin, *2hBG* 2-h postprandial blood glucose, *FBG* fasting blood glucose, *PIH* pregnancy-induced hypertension, *NICU* neonatal intensive care unit, *RR* relative risk, *CI* confidence interval, *MD* mean difference, *TM* telemedicine

In addition, we divided all the studies into four subgroups according to the patterns of TM interventions in the study; the subgroups consisted of group 1 (real-time monitoring and feedback), group 2 (health education and question answering), group 3 (real-time monitoring and feedback + health education and question answering) and group 4 (real-time monitoring and feedback + health education and question answering + peer support) (Table 4). Regarding the change in FBG, the change in 2hBG, and the incidence of caesarean section, PIH or preeclampsia, macrosomia, and preterm birth, the more comprehensive patterns of TM interventions (group 3 and 4) were indeed more effective than the simple patterns of TM interventions (group 1 and 2).

**Table 4:** Subgroup analyses of studies using different TM patterns

Outcome	TM pattern	No. of trials	Pooled effect			Heterogeneity	
			Effect size	95% CI	P	I^2^	P
Change in HbA1c	Group 1	5	MD = -0.34	(−0.67, −0.01)	0.04	94	< 0.01
	Group 2	3	MD = -0.63	(−1.11, −0.14)	0.01	83	< 0.01
Change in FBG	Group 1	8	MD = -0.25	(−0.43, −0.08)	< 0.01	56	0.03
	Group 2	4	MD = -0.29	(− 0.65, 0.06)	0.11	84	< 0.01
	Group 3	6	MD = -0.64	(−1.26, −0.02)	0.04	94	< 0.01
Change in 2hBG	Group 1	4	MD = -0.29	(−0.70, 0.11)	0.15	26	0.26
	Group 2	4	MD = -0.60	(−1.92, 0.72)	0.37	97	< 0.01
	Group 3	5	MD = -1.55	(−2.29, −0.81)	0.02	92	< 0.01
Caesarean section	Group 1	10	RR = 0.96	(0.73, 1.27)	0.77	65	< 0.01
	Group 2	3	RR = 0.67	(0.57, 0.92)	0.01	0	0.50
	Group 4	5	RR = 0.67	(0.57, 0.80)	< 0.01	0	0.73
PIH or preeclampsia	Group 1	7	RR = 0.69	(0.44, 1.08)	0.10	16	0.31
	Group 3	3	RR = 0.44	(0.35, 0.56)	< 0.01	0	0.98
Premature rupture of membranes	Group 1	4	RR = 0.76	(0.40, 1.44)	0.40	22	0.28
Macrosomia	Group 1	7	RR = 0.95	(0.55, 1.65)	0.85	32	0.19
	Group 2	5	RR = 0.43	(0.20, 0.92)	0.03	48	0.10
	Group 3	3	RR = 0.21	(0.09, 0.48)	< 0.01	61	0.08
	Group 4	3	RR = 0.44	(0.27, 0.71)	< 0.01	0	0.64
Neonatal hypoglycaemia	Group 1	7	RR = 1.20	(0.81, 1.78)	0.36	0	82.4
	Group 2	3	RR = 0.36	(0.22, 0.60)	< 0.01	0	0.78
Preterm birth	Group 1	7	RR = 0.42	(0.22, 0.80)	< 0.01	20	0.27
	Group 3	3	RR = 0.21	(0.13, 0.32)	< 0.01	16	0.30
Polyhydramnios	Group 3	3	RR = 0.17	(0.09, 0.32)	< 0.01	0	0.92
Admission to NICU	Group 1	7	RR = 0.88	(0.56, 1.40)	0.60	20	0.28
Neonatal jaundice or hyperbilirubinemia	Group 1	5	RR = 1.09	(0.69, 1.71)	0.71	0	0.72
NARDS	Group 1	4	RR = 0.81	(0.37, 1.77)	0.60	0	0.47

Group 1: real-time monitoring and feedback, Group 2: health education and question answering, Group 3: real-time monitoring and feedback + health education and question answering, Group 4: real-time monitoring and feedback + health education and question answering + peer support

*GDM* Gestational diabetes mellitus, *HbA1c* glycated haemoglobin, *2hBG* 2-h postprandial blood glucose, *FBG* fasting blood glucose, *PIH* pregnancy-induced hypertension, *NICU* neonatal intensive care unit, *RR* relative risk, *CI* confidence interval, *MD* mean difference, *TM* telemedicine

### Publication bias

**Fig. 4:**
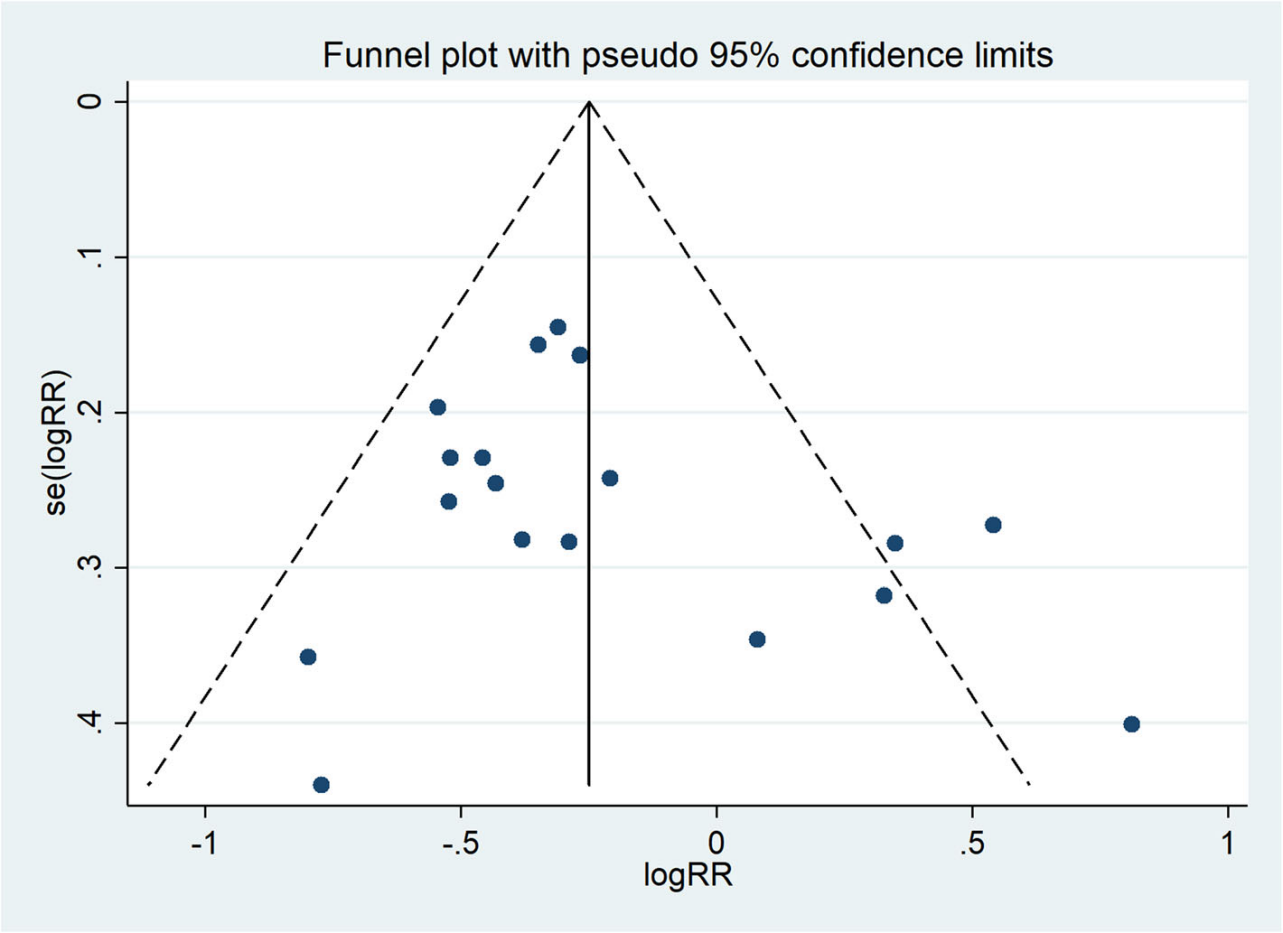
The funnel plot of the incidence of caesarean section

Funnel plots show that there might be publication bias in studies involving the incidence of caesarean section (Fig. 4), but no significant publication bias was found in the studies of the other 13 outcomes.

### Trial sequential analysis

**Fig. 5:**
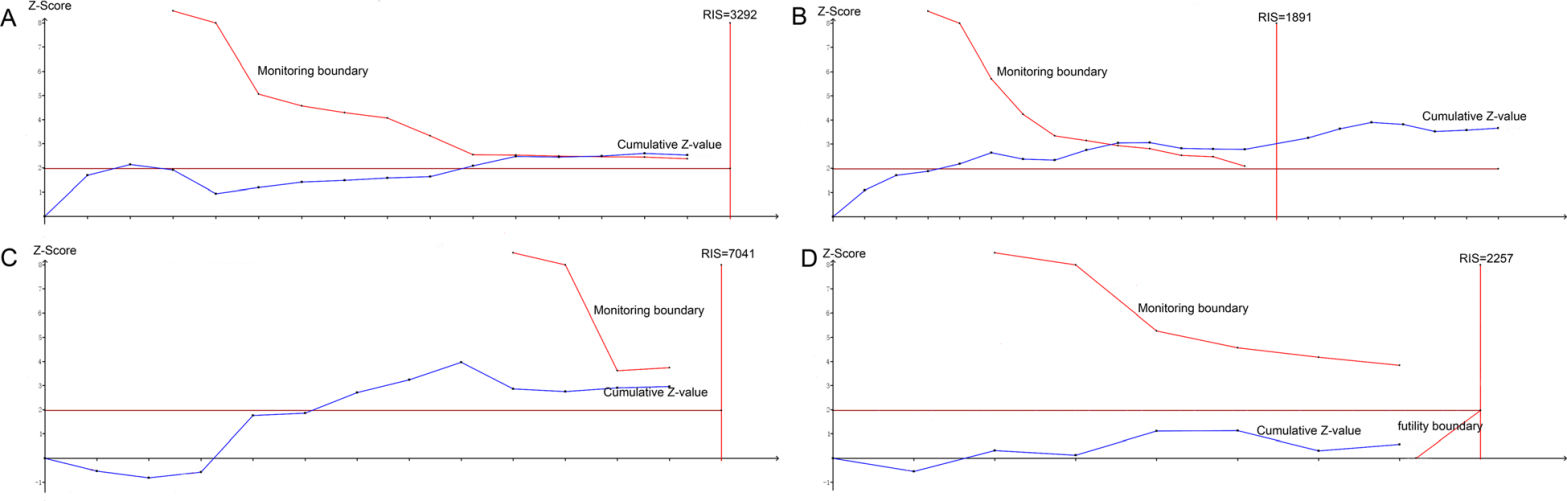
TSA results of the change in 2hBG (**a**), change in FBG (**b**), incidence of neonatal hypoglycaemia (**c**) and incidence of admission to NICU (**d**)

Figure 5a-d shows the TSA results of the change in 2hBG, the change in FBG, the incidence of neonatal hypoglycaemia and the incidence of admission to the NICU, respectively. The cumulative Z-value of the change in 2hBG surpassed the monitoring boundary, and the incidence of PIH or preeclampsia, premature rupture of membranes, premature birth, neonatal asphyxia, and polyhydramnios exhibited similar results. Moreover, the cumulative sample size of the change in FBG met the RIS which was similar to the change in HbA1c and the incidence of caesarean section. In addition, the cumulative Z values of the incidence of macrosomia and neonatal hypoglycaemia did not reach the monitoring boundary or the RIS line, and the cumulative Z values of the incidence of admission to the NICU, neonatal jaundice or hyperbilirubinemia, and NARDS did not surpass the futility boundary or the RIS.

## Discussion

The development of information and communications technology and the popularization of intelligent devices have resulted in a brand-new reform in medical treatment and clinical care. Increasing attention has been paid to the effectiveness of TM on GDM worldwide. This updated meta-analysis of 32 RCTs systematically evaluated the effectiveness of TM interventions compared with that of standard care on glycaemic control and pregnancy outcomes in patients with GDM. This study indicated that TM interventions can contribute to favourable impacts on HbA1c, FBG, and 2hBG. Regarding pregnancy outcomes, we found that TM interventions were inversely associated with the risk of caesarean section, PIH or preeclampsia, premature rupture of membranes, preterm birth, neonatal asphyxia, polyhydramnios, macrosomia, and neonatal hypoglycaemia. Moreover, the meta-analyses of studies involving the admission to the NICU, neonatal jaundice or hyperbilirubinemia and NARDS did not confirm significant differences between the two groups.

Two earlier meta-analyses on TM for the management of diabetes during pregnancy have previously been published. Ming et al. [14] carried out a meta-analysis of 7 RCTs with 579 pregnant women with any form of diabetes in pregnancy in 2016 and concluded that there was a significant difference in HbA1c between the TM group and the standard care group, and no effect was found in other maternal or neonatal outcomes. Rasekaba et al. [17] performed a meta-analysis of 3 RCTs involving 228 pregnant women with only GDM comparing TM to standard care in 2015 and found no beneficial impacts of TM on glycaemic control or pregnancy outcomes. By identifying and including additional recent trials with GDM only, the understanding of TM management of GDM was updated and more significant differences in maternal and neonatal/foetal complications were confirmed, which could provide scientific guidelines for future TM management. The greatest potential of TM lies in its ability to help patients who cannot be easily treated at existing clinics due to geographical constraints [50, 51].TM provides a convenient channel for communication between medical staff and patients, where medical staff could monitor the health status of patients in real-time, allowing patients to obtain individual treatment plans and disease-related knowledge of their own conditions, which may greatly improve the compliance of patients and further contribute to lower glycaemic levels [4, 14]. Some studies have shown that the psychological status of pregnant women also has a certain impact on pregnancy outcomes [52]. Timely communication between doctors and patients through the TM platform may reduce the anxiety of pregnant women and further reduce the risk of adverse outcomes on the basis of controlling the level of blood glucose.

Our finding on HbA1c was consistent with existing meta-analyses involving patients with type I diabetes and/or type II diabetes [8, 9, 53]. Marcolino et al. conducted a meta-analysis of 13 RCTs with 4207 diabetic patients and concluded that TM strategies were associated with improved HbA1c in patients with type I and II diabetes. Zhai et al. carried out a meta-analysis of 35 RCTs and found evidence of a reduction in HbA1c in the TM group among patients with type II diabetes mellitus. Wang et al. demonstrated that the TM intervention was inversely associated with the level of HbA1c in their meta-analysis involving a total of 602 type I diabetic patients. HbA1c is recognized as a key and valuable indicator of the effectiveness of treatment in diabetic patients, and a 1% reduction in HbA1c was associated with 21 and 37% risk reduction in diabetes-related death and microvascular complications, respectively, which would reduce the healthcare cost [54].

According to the results of meta-regression and subgroup analyses, the effectiveness of TM for GDM management may vary by the location of the study, the average age of pregnant women, the gestational age at enrolment, the proportion of primipara and the type of TM. The gestational age at enrolment reflected the period of the interventions, and earlier interventions could have led to a more significant change in HbA1c and 2hBG in this study, which illustrated the effect of TM to some extent. The location of the study may represent the inconsistency of scientific conditions and levels as well as various races of participants, resulting in heterogeneity among studies. Furthermore, the average age of pregnant women and the proportion of primipara can represent the physical and mental condition of pregnant women to some extent [55]. In addition, we found that WeChat interventions were more effective than other types of TM tools on more outcomes. Compared to other interventions, as a popular social app, WeChat was more convenient and accessible in daily life, and allowed patients to upload their glycaemic data, communicate and discuss with each other, reduce their psychological burden, enhance their confidence and receive more health information in many forms [16]. Considering the good operability and accessibility of social apps, it is believed that social apps will play essential roles in the clinical management of GDM in the future. Moreover, the subgroup analysis of the patterns of TM interventions demonstrated that the patterns of TM interventions may be another factor of the effectiveness. More comprehensive interventions could provide better management effects than simple interventions on most outcomes. The above results further suggested that what the TM is delivering or the strength of interventions is more important than the way it is delivered. And we should fully combine the characteristics of each TM tool and make full use of its functions to improve the management.

The results of TSA showed that cumulative Z-values of the change in HbA1c, 2hBG, and FBG, and the incidence of caesarean section, PIH or preeclampsia, premature rupture of membranes, premature birth, neonatal asphyxia, and polyhydramnios all surpassed the monitoring boundary or RIS line in TSA, which confirmed conclusive and sufficient evidence. In addition, cumulative Z-values of the incidence of macrosomia, neonatal hypoglycaemia, NICU, neonatal jaundice or hyperbilirubinemia, and NARDS suggest that the current evidence was not sufficient to fully indicate that the cumulative evidence reached a stable state and that further research on the comparison between TM and standard care is still needed to strengthen the evidence.

We embrace and appreciate that all scientific research has limitations, and our study had several to consider. Firstly, the standard care protocols and the diagnostic methods of GDM were not consistent among the included studies, which may have led to inconsistency and incomparability of the results. Secondly, random sequence generation methods and allocation concealment in most of the studies were not explicitly described which may have led to inaccurate assessment of research quality. Thirdly, blinding of healthcare providers and participants was not feasible in any of the included studies due to the nature of the TM intervention, and patients in the control group may obtain health information from other sources, which may cause biases in effect size. Finally, only literature published in Chinese and English were included in this study which may have resulted in the publication bias or language bias.

## Conclusions

In conclusion, telemedicine interventions contributed to beneficial impacts on the glycaemic level, and some maternal and neonatal/foetal complications in patients with gestational diabetes mellitus compared to the effects of standard care. The application of telemedicine in the clinical management of gestational diabetes mellitus may be advisable. Due to the limitation of systematic reviews and meta-analyses, an individual patient data meta-analysis or a well-designed randomized study may provide more important information for further management. Considering the high-speed development of information and communication technology and the complexity of gestational diabetes mellitus, the effectiveness of telemedicine for gestational diabetes mellitus needs to be further studied in the future.

## Supplementary information

**Additional file 1.** PRISMA checklist.

**Additional file 2.** The Web of Science search strategy.

**Additional file 3.** Detailed descriptions of TM interventions of included studies.

**Additional file 4: Table S1.** Additional subgroup analyses.

## Data Availability

The datasets used and/or analyzed during the current study are available from the corresponding author on reasonable request.

## Abbreviations

GDM: Gestational diabetes mellitus
HbA1c: glycated haemoglobin
2hBG: 2-h postprandial blood glucose
FBG: Fasting blood glucose
PIH: Pregnancy-induced hypertension
NICU: Neonatal intensive care unit
NARDS: Neonatal acute respiratory distress syndrome
RCT: Randomized controlled trials
TSA: Trial sequential analysis
RR: Relative risk
CI: Confidence interval
MD: Mean difference
TM: Telemedicine
RRR: Relative risk reduction
RIS: Required information size
CENTRAL: Cochrane Central Register of Randomized Control Trials
CNKI: Chinese National Knowledge Infrastructure
CBM: China Biology Medicine
