# Supplementary material for: Effectiveness of telemedicine for pregnant women with gestational diabetes mellitus: an updated meta-analysis of 32 randomized controlled trials with trial sequential analysis
Source: BMC Pregnancy Childbirth. 2020 Apr 6;20:198. doi: 10.1186/s12884-020-02892-1 (PMC7137255; doi:10.1186/s12884-020-02892-1)
Supplement: Supplementary file 2 — Additional file 2. The Web of Science search strategy. [file 12884_2020_2892_MOESM2_ESM.docx]

**The Web of Science search strategy is shown as follows:**

|  | **Area** | **Query** |
| --- | --- | --- |
| **#1** | **Telemedicine** | mobile OR digital OR mhealth OR m-health OR ehealthb OR e-health OR app OR apps OR tele-medicine OR tele-medicine OR smartphone OR smart phone OR cell phone OR telehealth OR tele-health OR tele-care OR telecare OR electronic* OR web-based OR technolog* OR Short messag* OR SMS OR remote OR internet OR Wechat OR QQ |
| **#2** | **Gestational diabetes** | pregnan* diabet* OR gestation* diabet* OR pregnan* hypergly* OR gestation* hypergly* OR GDM |
| **#3** | **Language** | English or Chinese |
| **#4** | **Time Limits** | From inception to 31^st^ July 2019 |
