# Supplementary material for: Effectiveness of telemedicine for pregnant women with gestational diabetes mellitus: an updated meta-analysis of 32 randomized controlled trials with trial sequential analysis
Source: BMC Pregnancy Childbirth. 2020 Apr 6;20:198. doi: 10.1186/s12884-020-02892-1 (PMC7137255; doi:10.1186/s12884-020-02892-1)
Supplement: Supplementary file 3 — Additional file 3. Detailed descriptions of TM interventions of included studies. [file 12884_2020_2892_MOESM3_ESM.docx]

**Additional Table** Detailed descriptions of TM interventions of included studies

| Study | TM interventions |
| --- | --- |
| Carral 2015 | Patients send BG data, insulin doses and other health data through the Web-based telemedicine system “DiabeTIC” and go to GDU every 6–8 weeks. The professionals monitored glucose control of patients every 2 weeks, and decide whether to maintain or modify the treatment of the patient. |
| Dalfra 2009 | Interfacing device “Glucopeep” allows conversion of BG values recorded by the glucometer into audio tones and transmission of glycaemic data through a telephone receiver. Doctors reviewed the data and transferred messages via the same system when a new message was available. Clinic visits once a month. |
| Given 2015 | Weekly review using telemedicine was considered as an addition to usual care. Use of TM hubs installed in patients’ home which would send the data of the previous 7 days to a central server and it would be accessed by TM providers. The professionals would contact pregnant women if the changes were needed. |
| Guo 2019 | Telemedicine was considered as an addition to usual care. Patients downloaded app “Dnurse”. Data for fasting and post-prandial glucose were monitored using the Dnurse BG meter then sent to the Doctors. Abnormal BG data would be noticed and analysed. The nurses provided online education available to patients every night. Additional knowledge about GDM could be learned by reading related education information in Dnurse App. |
| Homko 2007 | The web-based system “ITSMyHealthfile” was used to send health information at least 3 times per week and receive medical feedback from healthcare providers. |
| Homko 2012 | Internet-based system or phone was used to transmit the BG data weekly. Healthcare providers checked the data and gave feedback in time. |
| Mackillop 2018 | GDm-health app was used to upload the BG data and midwife reviewed the BG readings at least 3 times per week. The system would generate an alert when the data beyond the predefined thresholds. The patients could also receive a short message service message from nurses via the website. Clinic visits every 4 to 8 weeks. |
| Miremberg 2018 | Patients were asked to install an app in their mobile phone and document BG measurements via the app. Patients would receive individualized feedback from the healthcare team via e-mail every evening. |
| Perez-Ferre 2010 | Patients received a Glucometer and a cellular phone installing an app. The system allowed patients to upload the BG data regularly and contact with physicians through short text messages. |
| Rasekaba 2018 | Patients used a web-based system “Online Health Portfolio” to transmit the BG and related data and receive feedback from healthcare professionals. |
| Yang 2018 | Patients reported BG and related data via WeChat platform and healthcare providers issued targeted health and diet education diurnally. |
| Carolan-Olah 2019 | Health guidance for patients through Internet-based telemedicine, including diet and lifestyle education, and how to monitor the blood glucose levels. |
| Kim 2019 | Patients completed the online health diary once per week, transmitting daily FBG. The physicians reviewed dietary and lifestyle every week and conducted the individual education through the web-based system “DIETEX”. |
| Gao 2017 | Patients record the electronic data of BG with an app of mobile phone. The system will automatically judge the abnormal value and the doctor will feedback the corresponding health guidance in time through the data uploaded by the patients. |
| Hua 2018 | By setting up a WeChat official account, healthcare professionals answered questions from patients and issued health guidance through the official account. |
| Zhang 2018 | Patients upload BG data and related health data through the mobile nursing platform and asked questions online. Nurses would use the mobile platform to review the data and respond to questions from patients and feedback health guidance promptly. |
| Zhao 2018 | Patients upload BG data and related health data through the mobile nursing platform, and asked questions online. Nurses would use the mobile platform to review the data and respond to questions from patients and feedback health guidance on time. |
| Zeng 2017 | The clinical team set up a WeChat group and issued health knowledge about GDM every day, answering questions online regularly. And patients were encouraged to communicate with each other and report BG data in time. |
| Fang 2017 | The clinical team set up a WeChat group and issued health knowledge about GDM every day, answering questions online regularly. And patients were encouraged to communicate with each other and report BG data in time. |
| Ge 2017 | The researchers set up a WeChat group, regularly sent disease-related knowledge in the group, and actively encouraged patients in the group to communicate with each other. |
| Huang 2016 | Healthcare professionals set up a WeChat group and issued health knowledge about GDM every day, answering questions online regularly. |
| Jiang 2017 | Healthcare professionals set up a WeChat group, pregnant women in the group immediately raised related questions. Doctors and nurses regularly answer the question and publish medical nutrition therapy and exercise guidance in the WeChat Moments. |
| Jiang 2019 | Healthcare professionals added patients as their WeChat friends and conducted one-on-one personalized management for each patient. Meanwhile, WeChat groups were set up to share health knowledge and encourage patients to communicate with each other. |
| Jiang 2016 | Follow-up for patients was conducted by WeChat every 3 days, including carefully asking pregnant women's weight, blood pressure, blood glucose, etc., patiently answering related questions, providing dietary guidance and lifestyle guidance for pregnant women. |
| Liu 2018 | The patients uploaded BG and related data by using TM platform. The data were analysed and fed back to the patients in time, and the necessary guidance was given to the patients in terms of medicine, diet, exercise and so on. |
| Lu 2017 | Healthcare professionals set up a WeChat group and issued health knowledge about GDM every day, answering questions online regularly. Patients were required to upload food intake, weight, exercise, BG, collect and collate in real-time, and the data would be analysed and fed back to the patients in time, and the necessary guidance was given to the patients |
| Luo 2017 | WeChat platform was used to evaluate the nutrition status of pregnant women, understanding the lifestyles and nutrition knowledge and giving guidance to the unreasonable lifestyles and correct nutrition knowledge. And the WeChat platform was also used to record daily BG data, food intake. |
| Shao 2018 | Pregnant women added clinical dieticians as friends in WeChat. After the outpatient clinic, the dietitian sent the results of the 24-hour dietary survey to pregnant women through WeChat, comparing the results of the 24-hour dietary survey with the reference intake of dietary nutrients for Chinese residents. Clinical dieticians would ask them to follow the individual diet prescription. |
| Su 2018 | With the intervention of multiple TM management system, both mobile phones and computers can log in the website, which is directly connected to the hospital patient management platform, and the biochemical examination data of the patients can be recorded directly. Also, the doctors would provide psychological and physiological counselling after work in the QQ group and WeChat group. |
| Weng 2018 | The clinical team set up a WeChat group and a WeChat official account to regularly release relevant disease knowledge, healthy diet, and other contents and patients were encouraged to communicate with each other in the WeChat group. |
| Xiao 2016 | Senior nurse, who is in charge of WeChat official account, issued relevant disease information on a daily basis and gives corresponding guidance on patients' problems. |
| Yang 2015 | WeChat group was managed by doctors, dieticians, and nurses. Self-management knowledge is issued daily, including BG measurement, diet knowledge, exercise knowledge and so on. Pregnant women reported their status every day, and professionals give targeted guidance according to the report. |

**Note:** TM: telemedicine, BG: blood glucose, GDU: gestational diabetes unit.
