# Supplementary material for: Effectiveness of telemedicine for pregnant women with gestational diabetes mellitus: an updated meta-analysis of 32 randomized controlled trials with trial sequential analysis
Source: BMC Pregnancy Childbirth. 2020 Apr 6;20:198. doi: 10.1186/s12884-020-02892-1 (PMC7137255; doi:10.1186/s12884-020-02892-1)
Supplement: Supplementary file 4 — Additional file 4: Table S1. Additional subgroup analyses. [file 12884_2020_2892_MOESM4_ESM.docx]

**Table S1 Subgroup analyses of studies from China and non-China**

| Outcome | Country | No. of trials | Pooled effect | | |
| --- | --- | --- | --- | --- | --- |
|  |  |  | Effect size | 95%CI | P |
| Change in HbA1c | China | 9 | MD=-0.91 | (-1.32, -0.51) | <0.01 |
|  | Non-China | 3 | MD=-0.13 | (-0.30, 0.04) | 0.125 |
| Change in FBG | China | 15 | MD=-0.67 | (-1.04, -0.29) | <0.01 |
|  | Non-China | 5 | MD=-0.14 | (-0.26, -0.01) | 0.03 |
| Change in 2hBG | China | 14 | MD=-1.06 | (-1.91, -0.21) | 0.14 |
| Caesarean section | China | 11 | RR=0.75 | (0.61, 0.93) | 0.01 |
|  | Non-China | 8 | RR=0.92 | (0.68, 1.23) | 0.56 |
| PIH or preeclampsia | China | 7 | RR=0.43 | (0.35, 0.54) | <0.01 |
|  | Non-China | 5 | RR=0.85 | (0.49, 1.47) | 0.56 |
| Premature rupture of membranes | China | 5 | RR=0.64 | (0.49, 0.84) | <0.01 |
| Macrosomia | China | 13 | RR=0.34 | (0.22, 0.55) | <0.01 |
|  | Non-China | 5 | RR=1.37 | (0.88, 2.12) | 0.16 |
| Neonatal hypoglycaemia | China | 7 | RR=0.42 | (0.29, 0.62) | <0.01 |
|  | Non-China | 5 | RR=1.28 | (0.85, 1.91) | 0.23 |
| Preterm birth | China | 8 | RR=0.26 | (0.17, 0.40) | <0.01 |
|  | Non-China | 5 | RR=0.60 | (0.32, 1.14) | 0.12 |
| Neonatal asphyxia | China | 5 | RR=0.17 | (0.08, 0.33) | <0.01 |
| Polyhydramnios | China | 6 | RR=0.17 | (0.10, 0.29) | <0.01 |
| Admission to NICU | Non-China | 6 | RR=0.93 | (0.57, 1.53) | 0.78 |
| Neonatal jaundice or hyperbilirubinemia | Non-China | 4 | RR=1.17 | (0.69, 1.98) | 0.56 |
| NARDS | Non-China | 4 | RR=0.81 | (0.37, 1.77) | 0.60 |

**Table S2 Subgroup analyses of Chinese studies using WeChat and non-WeChat**

| Outcome | TM tools | No. of trials | Pooled effect | | |
| --- | --- | --- | --- | --- | --- |
|  |  |  | MD | 95%CI | P |
| Change in HbA1c | WeChat | 6 | -0.84 | (-1.46, -0.22) | <0.01 |
|  | Non-WeChat | 3 | -1.03 | (-1.57, -0.48) | <0.01 |
| Change in FBG | WeChat | 9 | -0.85 | (-1.35, -0.35) | <0.01 |
|  | Non-WeChat | 6 | -0.39 | (-1.02, 0.24) | 0.23 |
| Change in 2hBG | WeChat | 8 | -1.26 | (-2.63, 0.11) | 0.07 |
|  | Non-WeChat | 6 | -0.86 | (-1.75, 0.03) | 0.06 |
